# Viral Etiology of Acute Gastroenteritis Among Forcibly Displaced Myanmar Nationals and Adjacent Host Population in Bangladesh

**DOI:** 10.1093/infdis/jiab466

**Published:** 2021-09-29

**Authors:** Mohammad Enayet Hossain, Md Muzahidul Islam, Mojnu Miah, Warda Haque, Jan Vinjé, Mohammed Ziaur Rahman, Abu Syed Golam Faruque, Azharul Islam Khan, Tahmeed Ahmed, Mustafizur Rahman

**Affiliations:** 1 International Centre for Diarrhoeal Disease Research, Bangladesh, Dhaka, Bangladesh; 2 Centers for Disease Control and Prevention, Atlanta, Georgia, USA

**Keywords:** AHP, Bangladesh, Viral etiology, diarrhea, FDMN

## Abstract

**Background:**

Since August 2017, Myanmar nationals from Rakhine state have crossed the border into Bangladesh and settled in Cox’s Bazar, the World’s largest refugee camp. Due to overcrowding, poor sanitation, and hygienic practices they have been under significant health risks including diarrheal diseases. The objective of this study is to determine the viral etiology of acute gastroenteritis (AGE) among forcibly displaced Myanmar nationals (FDMN) and adjacent Bangladeshi local host population (AHP).

**Methods:**

From April 2018 to April 2019, we collected stool specimens from 764 FDMN and 1159 AHP of all ages. We tested 100 randomly selected specimens from each group for the most common AGE viruses.

**Results:**

Among 200 diarrhea patients, 55% and 64% of FDMN and AHP patients, respectively, had viral infections; the most common viruses were rotavirus (29% vs 44%), adenovirus (24% vs 31%), and norovirus (14% vs 10%). In both populations, viral infections were significantly higher in children less than 5 years of age, compared with bacterial infections that were higher in patients older than 5 years of age (*P* ≤ .05).

**Conclusions:**

Disparities in viral and bacterial prevalence among various age groups warrant careful antibiotic usage, especially in children less than 5 years of age.

The Rohingya people are one of the most persecuted minorities in the world and reside in Rakhine state, Myanmar [1]. These denied citizens are restricted from access to health, education, and childhood vaccinations [2]. Bangladesh has been hosting forcibly displaced Myanmar nationals (FDMN) since 1978. In 2017–2018, violence in the Rakhine State of Myanmar prompted a massive influx of FDMN (~700000) to the bordered district of Cox’s Bazar, Bangladesh, joining the approximately 213000 Rohingya who had fled in earlier influxes [1, 3–5]. Several international humanitarian organizations, local governments, and nongovernmental organizations have been providing temporary shelters, access to sanitation, and healthcare. When additional care is required, patients are transferred to local governmental medical college hospitals in Cox’s Bazar and Chittagong [6–8]. In the Cox’s Bazar Rohingya camp, overcrowding FDMN lack access to healthcare, nutrition, safe water, and proper sanitation. These factors make them vulnerable to infectious diseases and increase the risk of an acute diarrheal outbreak, especially among children below 5 years of age [5, 9]. In addition to reported outbreaks of diphtheria, measles, and varicella [10–12], acute gastroenteritis (AGE) remains a constant threat among the FDMN.

Acute gastroenteritis remains a major cause of morbidity and mortality in children <5 years of age in low- and middle-income countries and accounts for 31% of deaths in the South-East Asian region [13]. Viruses such as rotavirus group A, norovirus, adenovirus, astrovirus, and sapovirus are established etiological agents. In Bangladesh, among children <5 years of age, the most prevalent etiological agents are group A rotavirus [14, 15], norovirus [16, 17], and less prevalent enteric adenovirus [18], astrovirus [19], and sapovirus [20]. However, in the Rakhaine state of Myanmar, the published data on the viral etiology of acute gastroenteritis is either limited or unavailable. Only data on rotavirus gastroenteritis are available in Myanmar, and the most recent reported data ranged from 42% to 56% among children under 5 [21].

In the world’s largest refugee camp in Cox’s Bazar, the prevalence of diarrheal diseases was reported significantly high [5, 22]. According to the United Nations (UN) Registered Myanmar Nationals (URMN) morbidity and mortality reports, people are exhausted, and children under 5 years are at high risk of diarrhea and cholera outbreaks [5]. Moreover, in the unregistered Rohingya camp, high population density, inadequate health facilities, and open defecation increase diarrheal disease risk [23, 24]. The morbidity of diarrheal disease in the refugee camps accounted for 7%–9% of the reported cases, and EWARS reported many cases of bloody diarrhea [7, 25]. Despite the high risk of diarrheal disease among the Rohingya population, no etiological investigations were conducted. Identifying specific etiologies of AGE is necessary to target potential measures, such as vaccinations. In the current study, we investigated viral etiologies of AGE among FDMN and adjacent Bangladeshi local host population (AHP) in Cox’s Bazar, Bangladesh.

## MATERIALS AND METHODS

### Ethical Statement

This study was approved by the institutional review board (Research Review Committee and Ethical Review Committee [protocol no. PR-17111]) of the International Centre for Diarrhoeal Disease Research, Bangladesh (icddr,b). Written informed consent was obtained from the participants or from parents or guardians, and assent was also obtained from participants who were 11 to 17 years of age before enrollment. Assurance was given to the participants about the nondisclosure of personal information such as their names or identity, and the data will be used for improving patient care activities such as publication.

### Study Setting

The study was conducted in Ukhia upazilla of Cox’s Bazar district, where the displaced Myanmar nationals settled. Diarrhea patients from FDMN and AHP of all ages who visited an icddr,b-operated diarrhea treatment center were enrolled in the study.

### Sample and Data Collection

Stool specimens were collected during April 2018–April 2019. A total of 764 diarrheal stool specimens from FDMN and 1159 from AHP of all ages were collected. A subset of 100 specimens for each group was randomly selected for etiology investigation. Demographic and clinical information of the diarrheal patients were obtained from the icddr,b surveillance system.

### Ribonucleic Acid Extraction and Real-Time Reverse-Transcriptase Polymerase Chain Reaction

The InviMag Pathogen kit (STRATEC Molecular GmBH, Berlin, Germany) was used for ribonucleic acid (RNA) extracted from each stool sample on an automatic extractor (Flex 96; KingFisher). Duplex real-time reverse-transcriptase polymerase chain reaction (rRT-PCR) assay was performed to detect GI/GII norovirus with primers and probes described previously [26, 27]. All specimens were also screened for the following viral pathogens: group A rotavirus, adenovirus, astrovirus, and sapovirus using rRT-PCR as described elsewhere [28–31]. The real-time (RT-)PCR assays were carried out in a 25-μL reaction mixture consisting of 12.5 μL Ag-Path buffer (Ambion Inc., Austin, TX),1 μL Ag-Path enzyme mix, 0.8 pmol of each forward and reverse oligonucleotide primer, 0.2 pmol of each oligonucleotide probe, and 5-μL RNA template. The PCR condition was as follows: reverse transcription 55°C for 30 minutes, denaturation at 95°C for 30 seconds, and then 45 cycles of denaturation at 95°C for 15 seconds and annealing-extension at 60°C for 1 minute.

### Conventional Reverse-Transcriptase Polymerase Chain Reaction and Sequencing

Norovirus genotyping was done by amplifying a short overlapping region of the ORF1-ORF2 by conventional RT-PCR using the QIAGEN OneStep RT-PCR Kit (QIAGEN, Hilden, Germany) as described previously [32]. Rotavirus genotyping was done using multiplex RT-PCR as described elsewhere [33–35]. The amplified PCR products were purified with the ExoSAP-IT PCR product cleanup kit (Affymetrix, Inc., Cleveland, OH) and sequenced using BigDye Terminator kit (Perkin-Elmer Applied Biosystems, Foster City, CA) in an automated genetic analyzer ABI 3500xL (Perkin-Elmer Applied Biosystems).

### Data Analysis

Clinical symptoms such as duration of diarrhea, fever, abdominal pain, vomiting, and dehydration status in FDMN were compared with the AHP using Fisher’s exact 2-sided test, or χ^2^ test, as appropriate. The strength of association was determined by estimating odds ratio (crude OR) and its 95% confidence interval (CI). The Statistical Package for Social Sciences (SPSS) for Windows (version 20.0; Chicago, IL) and Epi Info (version 7.1; Centers for Disease Control and Prevention, Atlanta, GA) were used to carry out data analysis. Norovirus genotype was determined using web-based genotyping tool https://norovirus.ng.philab.cdc.gov/ [36], and rotavirus genotyping was done using the online rotavirus genotyping tool, Rota C (http://rotac.regatools.be/) [37].

## RESULTS

**Figure 1.**
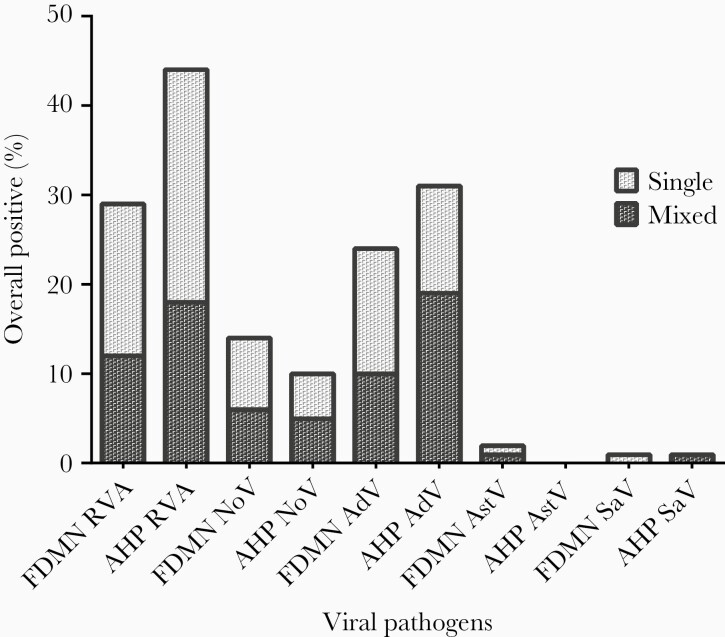
Prevalence of viral pathogens among forcibly displaced Myanmar nationals (FDMN) and adjacent Bangladeshi local host population (AHP). AdV, adenovirus; AstV, astrovirus; NoV, norovirus; RVA, rotavirus; SaV, sapovirus.

Of the 100 FDMN and 100 AHP with diarrhea, 55% and 51% were males, and 49% and 64% were less than 5 years of age (U5 children), respectively (Table 1). A total of 55% of FDMN and 64% of AHP tested positive for at least 1 enteric virus (OR = 0.689; 95% CI, 0.39–1.21; *P* = .25). Viral coinfections were detected in 14% of FDMN and 21% of AHP. The most common virus (single and coinfection) was rotavirus with 29% in FDMN and 44% in AHP (OR = 0.52; 95% CI, 0.29–0.933; *P* ≤ .05), followed by human enteric adenovirus, 24% vs 31%, respectively, in FDMN and AHP (OR = 0.703; 95% CI, 0.38–1.313; *P* = .34), and norovirus, 14% vs 10%, respectively (OR = 1.465; 95% CI, 0.62–3.475; *P* = .51) (Table 2). These top 3 viruses together constituted 98% of all viruses (Figure 1). The most frequently detected virus as a single infection among FDMN was rotavirus (17%), followed by adenovirus (14%), and norovirus (8%). Among the AHP, the most frequently detected single infection was rotavirus (26%), followed by adenovirus (12%) and norovirus (5%) (Figure 1). The most frequently detected coinfection among both populations was rotavirus and adenovirus (Supplementary Table 1).

**Table 1.** Baseline Demographic and Clinical Characteristics of the FDMN and AHP Included in the Univariate Analysis

Variables	FDMN	AHP
	N = 100, % (n)	N = 100, % (n)
Age in year (Years, Mean ± SD)	14.6 ± 3.7	13.7 ± 4.4
Sex (%; n/total)		
Male	55.0 (55)	51.0 (51)
Female	45.0 (45)	48.0 (48)
Transgender	0.0 (0)	1.0 (1)
Treatment of diarrheal diseases taken before coming to the hospital. ORS (%; n/total)	65.0 (65)	72.0 (72)
Antibiotic (oral)	14.0 (14)	27.0 (27)
Antibiotic (injectable)	0.0 (0)	1.0 (1)
IV at home / Other health care	3.0 (3)	3.0 (3)
Unknown Medicine	11.0 (11)	9.0 (9)
Duration of diarrhea before arrival at Hospital		
<1 Day	26.0 (26)	34.0 (34)
1-3 Days	67.0 (67)	56.0 (56)
4-6 Days	7.0 (7)	10.0 (10)
Character of stool		
Watery	53.0 (53)	53.0 (53)
Rice watery	9.0 (9)	22.0 (22)
Semisolid	24.0 (24)	18.0 (18)
Mucous mixed	13.0 (13)	7.0 (7)
Blood + Mucous	1.0 (1)	0.0 (0)
Number of stool in 24 hours (mean ± sem)	12.8 ± 0.61	13.6 ± 0.72
Abdominal Pain	43.0 (43)	53.0 (53)
Number of vomiting in last 24 hours		
No	37.0 (37)	28.0 (28)
<10	47.0 (47)	57.0 (57)
≥10	16.0 (16)	15.0 (15)
Dehydration status		
No Sign	55.0 (55)	63.0 (63)
Some	40.0 (40)	32.0 (32)
Severe	5.0 (5)	5.0 (0)
Eye (Dehydration status)		
Normal	80.0 (80)	77.0 (77)
Sunken	20.0 (20)	23.0 (23)
Mouth/Tongue (Dehydration status)		
Normal	58.0 (58)	68.0 (68)
Somewhat dry	38.0 (38)	29.0 (29)
Very Dry	4.0 (4)	3.0 (3)

Abbreviations: AHP, adjacent Bangladeshi local host population; FDMN, forcibly displaced

Myanmar nationals; IV, intravenous; ORS, oral rehydration solutions; SD, standard deviation;

sem, standard error of the mean.

**Table 2.** Prevalence of Enteric Viral Pathogens Among FDMN and AHP

Viral Pathogens	FDMN^a^ (%) (n = 100)	AHP^a^ (%) (n = 100)
Rotavirus	29 (29)	44 (44)
Norovirus	14 (14)	10 (10)
Adenovirus	24 (24)	31 (31)
Sapovirus	1 (1)	1 (1)
Astrovirus	2 (2)	0
At least 1 viral pathogen	55 (55)	64 (64)

Abbreviations: AHP, adjacent Bangladeshi local host population; FDMN, forcibly displaced Myanmar nationals.

a Data are number of total infections.

Bacterial pathogens were isolated from 17% FDMN and 19% AHP. The prevalence of bacterial infections was significantly higher among patients above 18 years of age in FDMN (32.5%) (OR = 6.741; 95% CI, 2.008–22.63; *P* = .001) and patients of >5–18 years of age in the AHP (57.1%) (OR = 6.933; 95% CI, 1.406–34.19; *P* ≤ .05) (Supplementary Table 2).

### Clinical Features of Acute Gastroenteritis Cases

The AGE symptoms of both FDMN and AHP were compared to observe whether patients were infected with a single pathogen or coinfected with other enteric pathogens (Table 1). No significant differences in clinical manifestations were noticed between positive or negative cases and patients with single or coinfection. Of the FDMN, major clinical manifestations were vomiting (63%), abdominal pain (43%), and moderate to severe dehydration (45%). Among AHP, almost similar clinical symptoms were observed: vomiting (72%), abdominal pain (53%), and moderate to severe dehydration (37%). The FDMN patients had a lower frequency of defecation (*P* < .001) and watery rice stool (*P* = .01) than the AHP (Table 1). In both populations, the patients infected with norovirus were more dehydrated (*P* < .001), had a higher frequency of stool per day and vomiting, and less abdominal pain compared with patients infected with rotavirus. Frequencies of stools and vomiting were higher in rotavirus-infected patients and had less abdominal pain than patients with adenovirus. Patients infected with bacteria had abdominal pain (37%), vomiting (58%), and severe dehydration (21%) (Table 3). As part of AGE treatment, both FDMN and AHP patients (14% vs 28%) were given antibiotics (oral and/or injection) (Table 1); however, 76% of the treated patients had no evidence of bacterial infections.

**Table 3.** Comparison of Clinical Symptoms Associated With Single and Coinfections of Viral and Bacterial Pathogens in FDMN and AHP

Variables	Rotavirus (n = 43)		Norovirus (n = 13)		Adenovirus (n = 26)		Sapovirus (n = 1)		Astrovirus (n = 1)		Viral co-infection (n = 35)		Bacterial pathogen (n = 19)	
	FMDN (n = 17)	AHP (n = 26)	FMDN (n = 8)	AHP (n = 5)	FMDN (n = 14)	AHP (n = 12)	FMDN (n = 1)	AHP (n = 0)	FMDN (n = 1)	AHP (n = 0)	FMDN (n = 14)	AHP (n = 21)	FMDN (n = 11)	AHP (n = 8)
Sex														
Male	9	11	4	3	6	5	0	0	0	0	11	12	5	3
Female	8	15	4	2	8	7	1	0	1	0	3	8	6	5
Transgender	0	0	0	0	0	0	0	0	0	0	0	1	0	0
Duration of diarrhea before arrival at Hospital														
<1 days	5	8	3	2	4	4	0	0	1	0	3	5	5	5
1-3 days	11	13	5	3	10	7	1	0	0	0	7	14	6	3
4-6 days	1	5	0	0	0	1	0	0	0	0	4	2	0	0
Diarrhea (in last 24h)														
≤10 times daily	10	12	2	1	6	6	0	0	0	0	5	7	5	1
>10 times daily	7	14	6	4	8	6	1	0	1	0	9	14	6	7
Vomiting (in last 24h)														
No	5	7	2	0	7	8	0	0	0	0	5	1	6	2
<10	12	18	4	5	5	4	1	0	1	0	5	13	4	3
≥10	0	1	2	0	2	0	0	0	0	0	4	7	1	3
Dehydration														
No sign	15	19	2	2	9	11	0	0	1	0	10	13	6	2
Some	2	7	5	2	5	1	1	0	0	0	4	8	4	3
Severe	0	0	1	1	0	0	0	0	0	0	0	0	1	3
Abdominal pain														
Yes	5	11	0	3	5	6	1	0	1	0	5	8	5	2
No	12	15	8	2	9	6	0	0	0	0	9	13	6	6
Character of stool														
Watery	11	13	6	4	9	6	1	0	1	0	4	13	6	3
Rice watery	2	6	1	0	0	3	0	0	0	0	3	7	1	2
Semisolid	3	6	1	1	3	3	0	0	0	0	6	1	3	1
Mucus mixed	1	1	0	0	2	0	0	0	0	0	1	0	1	2

Abbreviations: AHP, adjacent Bangladeshi local host population; FDMN, forcibly displaced Myanmar nationals.

### Age Group Distribution

**Figure 2.**
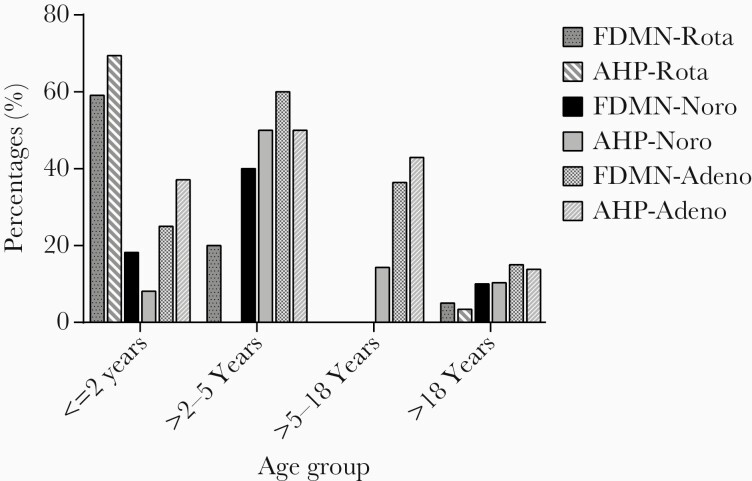
Distribution of viral pathogens among different age groups of forcibly displaced Myanmar nationals (FDMN) and adjacent Bangladeshi local host population (AHP). Adeno, adenovirus; Noro, norovirus; Rota, rotavirus.

**Figure 3.**
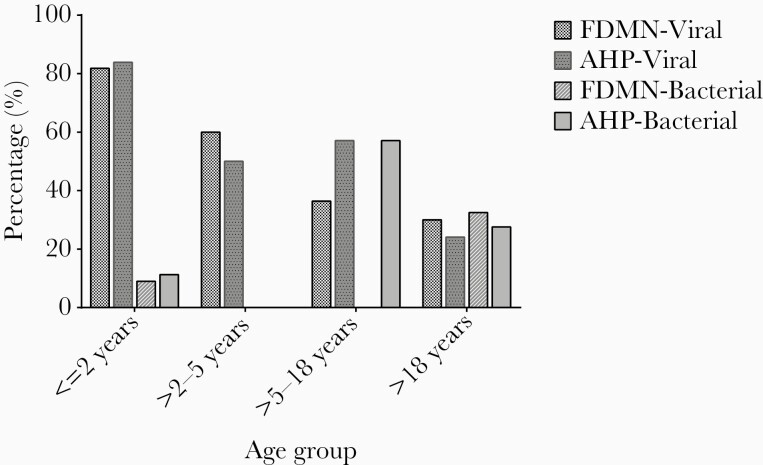
Comparison of the prevalence between viral and bacterial enteric pathogens among forcibly displaced Myanmar nationals (FDMN) and adjacent Bangladeshi local host population (AHP) in different age groups.

The subjects were categorized into 4 age groups (≤2 years, >2–5 years, >5–18 years, and >18 years). The majority of viral infections were detected in U5 children in FDMN (71) and AHP (83%) (Figure 2). The highest prevalence of bacterial infection was detected in >18 years of age group in FDMN (33%), whereas in the >5- to 18-year-old age group (57%) the highest prevalence was in AHP (Figure 3). Rotavirus A infection was predominant in children of the ≤2 years age group: the prevalence was 59% for FDMN (OR = 25.52; 95% CI, 6.891–94.5; *P* < .01) and 69% in AHP (OR = 83.74; 95% CI, 10.69–655.8; *P* < .01) (Figure 2). Among the FDMN children of >2–5 years, the rate of rotavirus infection was 20%, absent among children 5–18 years, and less prevalent in adults more than 18 years (5%). The highest rate of norovirus infection was found in children 2–5 years of age (40% vs 50%), followed by ≤2 years (19% vs 8%), >5–18 years (0% vs 14%), and >18 years (10% vs 10%) age groups, respectively, for FDMN and AHP. Of note, 71% of all positive norovirus cases were U5 children among FDMN (OR = 6.25; 95% CI, 1.213–32.21; *P* < .05), whereas 60% of positive cases were detected in children within the same age group in AHP. The prevalence of enteric adenovirus was lower in FDMN than AHP irrespective of all age groups except children >2–5 years (Figure 2). Among AHP adenovirus was frequently detected in U5 children (OR = 2.49; 95% CI, 0.94–6.55; *P* < .05), but no significant difference in age groups was found in FDMN.

### Rotavirus Genotype Distribution

**Figure 4.**
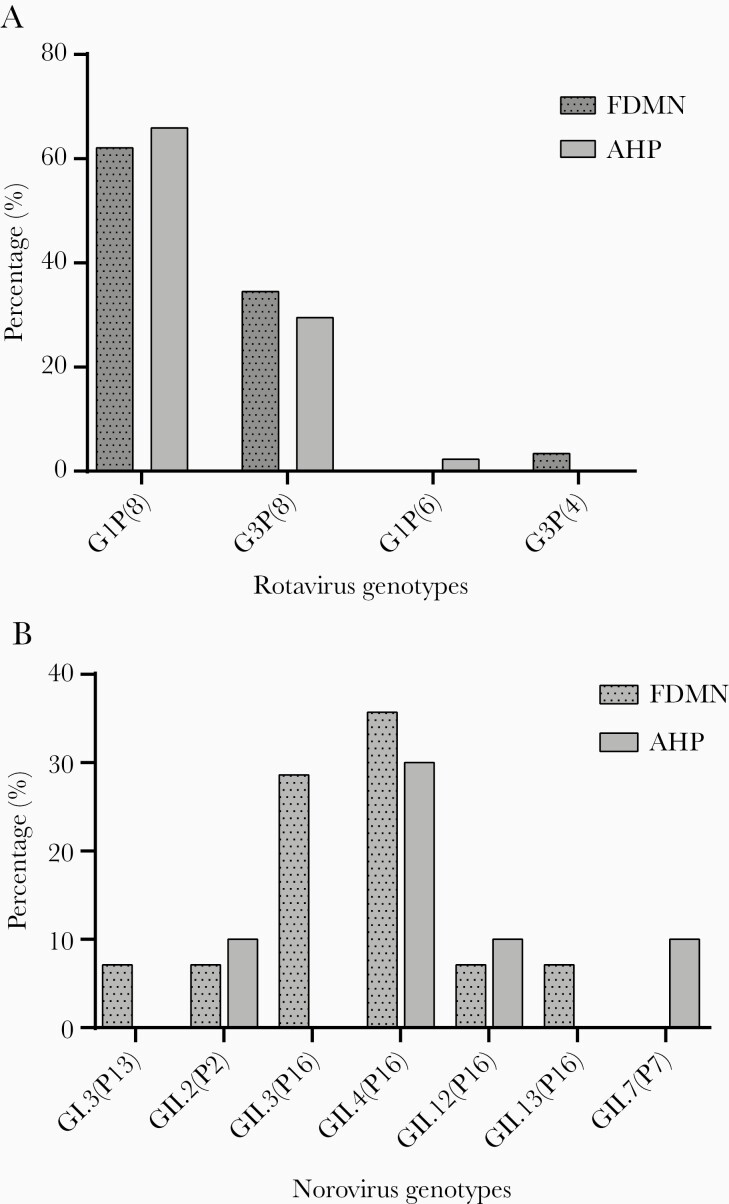
Genotype distribution of of rotavirus (A) and norovirus (B) among forcibly displaced Myanmar nationals (FDMN) and adjacent Bangladeshi local host population (AHP).

Among FDMN and AHP, the most prevalent rotavirus genotype was G1P[8] (62% and 66%, respectively), followed by G3P[8] (35% and 30%, respectively). Single infection with G3[P4] (3%) and G1P[6] (2%) was detected in FDMN and AHP. For P genotypes, almost all strains were P[8]; 97% in FDMN and 98% in AHP (Figure 4A).

### Norovirus Genotype Distribution

Norovirus genogroup II viruses were the most predominant (93% in FDMN and 70% in AHP). The globally dominant GII.4[P16] genotype was frequently identified in both populations (36% in FDMN and 30% in AHP). Other common genotypes include GII.3[P16] and GII.2[P2] in FDMN and GII.13[P16], GII.2[P2], and GII.7[P7] in AHP (Figure 4B). A novel norovirus recombinant genotype, GII.12[P16], was identified in both populations. It is interesting to note that one recombinant genotype GII.3[P16] was a major norovirus genotype in FDMN (29%) but was absent in AHP.

## DISCUSSION

We report the viral etiology of AGE among the FDMN and local host population (AHP) residing in the Cox’s Bazar district adjacent to the Rohingya camp. This study was conducted on reported gastroenteritis patients of both populations. Our data highlight the prevalence of common enteric viruses and important contributions to the etiology of AGE among the participants. Although the prevalence of the individual viruses was slightly different, the overall burden of viruses in AGE was comparable between the 2 populations. Among the tested patients, AGE agents (virus and bacteria) were detected in 72% of FDMN and 80% of AHP. Negative patients had similar symptoms; they could have infected with other viruses related to gastroenteritis, such as human parechovirus, bocavirus, aichivirus, and/or other bacteria, which were not investigated.

Viral infections were significantly higher among children ≤2 years in FDMN and AHP, and the rate decreased in the higher age groups (Figure 4). In contrast, bacteria were responsible for a higher proportion of diarrhea in patients older than 5 years and significantly higher in patients >18 years of age in agreement with a previous study [38].

The rate of rotavirus infections was lower in FDMN than in AHP. However, the prevalence in FDMN U5 children (55%) was much higher than previously reported in Myanmar [21]. Similarly, the prevalence in AHPU5 children was also higher (67%) than previously reported in Bangladesh [14, 15] and neighboring countries such as China, India, Indonesia, Malaysia, South Korea, and Taiwan [39, 40]. Rotavirus infections were absent in the 5- to 18-year-old age group of both populations and less prevalent in adults, comparable to other studies in Bangladesh [14, 15]. Among rotaviruses, G1P[8] was the most prevalent genotype in both FDMN and AHP, which is higher than previously reported in Myanmar during 2009–2016 [41] and which was the predominant genotype in Bangladesh from 2002 to 2016 [14]. Over the last 17 years in Bangladesh and Myanmar, the most frequently identified rotavirus genotypes G2, G9, and G12 were completely absent in both populations. It is remarkable that G3 viruses replaced G2 and G12 genotypes, and it became the second most prevalent genotype in both populations. In Bangladesh, G3 viruses emerged among rhesus macaques in 2013 [42] and in humans in 2016 [14]. To our knowledge, infections with the G3P[4] genotype identified in FDMN was absent in Bangladesh and Myanmar since 2006 [41, 43].

The rate of norovirus infections was slightly higher in FDMN than AHP; however, it was lower than previously reported in Bangladesh [16, 17]. The prevalence of norovirus infection among U5 children in FDMN was comparable to the global prevalence, which was higher than AHP [17, 44]. Some of the clinical manifestations observed in this study related to norovirus infection, such as number of defecation and frequency of vomiting, were much higher than reported previously in Bangladesh [16, 17]

The genotype distribution of noroviruses was different between FDMN and AHP. It is interesting to note that GII.3[P16] viruses were detected among 28.5% of the FDMN patients but were not detected in the AHP host population. A novel recombinant norovirus GII.12[P16] genotype, which was never reported from Bangladesh, was detected in both populations. This novel strain was associated with epidemic and endemic gastroenteritis in hospital settings in Alberta, Canada, during the 2018–2019 season [45].

Enteric adenoviruses were the second most prevalent viruses detected in both FDMN and AHP. The overall prevalence of adenovirus was significantly higher in FDMN and AHP than reported previously [18]. The detection rate was 6 to 12 times higher than the previous studies conducted in Bangladesh [46]. The rate of adenovirus infection among all age groups in both populations were remarkable compared with previous studies conducted worldwide, such as India, Japan, China, Thailand, and Bangladesh [18, 47, 48].

The prevalence of astrovirus and sapovirus was very low in both populations, similar to previous reports in children less than 2 years old [19, 20]. Most of the children infected with astrovirus and sapovirus in this study also tested positive for ≥1 other enteric virus or bacteria.

Twenty-nine percent of the patients with AGE were coinfected with multiple viruses. This finding is in agreement with previous reports [16, 17, 49]. Several reports have suggested that coinfection with different enteric pathogens may be responsible for disease severity in gastroenteritis [50]. Viral coinfections included combinations of rotavirus-adenovirus and rotavirus-norovirus, which were not reported previously [16–18]. Most of the coinfections (89%) were detected in children ≤2 years of age [49].

The FDMNs are exposed to significant public health risks due to overcrowded living conditions and compromised water, sanitation, and hygiene (WASH) practices at the camps, and they are supposed to have a much higher frequency of viral pathogens associated with AGE. However, our results indicated that they were diagnosed with a similar frequency of gastroenteritis viruses compared with the adjacent local populations, which was not unexpected due to several reasons. First, we found that the water sources of both populations were almost similar; both populations used water from tube wells, public taps, boreholes, hand pumps, tap stand/piped water, water tank, and protected and unprotected dug well (data not shown). Second, we observed a (semi)-controlled lifestyle of the refugees inside the camp; many national and international organizations are continuously working on the water quality and providing hygienic practices in the camp. In contrast, hygienic practice at the surrounding local areas to the camp has lacked monitoring and surveillance. The similar yet high vulnerability of both populations to viral gastroenteritis warrants a vaccination campaign against rotaviruses in both communities.

We observed misuse of antibiotics as a treatment of diarrhoeal diseases in both populations, although they had a lower frequency of bacterial pathogens. Previous studies have demonstrated that a tendency of self-medication, antibiotic misuse, and treatment of nonbacterial illness can instinctively incite proliferation of antibiotic resistance and generation of multidrug-resistant microorganisms [51–54].

## CONCLUSIONS

In conclusion, our findings of the etiology of AGE among the FDMN and AHP may help with the management of these frequently detected cases of gastroenteritis in displaced people. The high prevalence of viral infections in children U5 children, especially rotavirus, suggests the importance of the implementation of mass vaccination among FDMN and AHP. Disparities in viral and bacterial prevalence among various age groups warrant careful antibiotic usage, especially in U5 children.

## Supplementary Data

Supplementary materials are available at *The Journal of Infectious Diseases* online. Consisting of data provided by the authors to benefit the reader, the posted materials are not copyedited and are the sole responsibility of the authors, so questions or comments should be addressed to the corresponding author.

jiab466_suppl_Supplementary_Table_S1

jiab466_suppl_Supplementary_Table_S2

## Notes

***Acknowledgments.*** The International Centre for Diarrhoeal Disease Research, Bangladesh (icddr,b) acknowledges with gratitude the commitment of UNICEF, Bangladesh for their continuous support. The icddr,b is also grateful to the Governments of Bangladesh, Canada, Sweden, and the United Kingdom for providing core/unrestricted support.

***Financial support.*** This research protocol was funded by UNICEF.

***Potential conflicts of interest.*** All authors: No reported conflicts of interest. All authors have submitted the ICMJE Form for Disclosure of Potential Conflicts of Interest.
