# Supplementary material for: Viral Etiology of Acute Gastroenteritis Among Forcibly Displaced Myanmar Nationals and Adjacent Host Population in Bangladesh
Source: J Infect Dis. 2021 Sep 29;224(Suppl 7):S864–72. doi: 10.1093/infdis/jiab466 (PMC8687051; doi:10.1093/infdis/jiab466)
Supplement: jiab466_suppl_Supplementary_Table_S1 [file jiab466_suppl_supplementary_table_s1.docx]

| **Table S1: Co-infections of viral pathogen in acute gastroenteritis** | | |  |  |  |
| --- | --- | --- | --- | --- | --- |
| **Co-infection** | **FDMN (%)** | **AHP (%)** |  |  |  |
| RVA+AdV | 7 | 16 |  |  |  |
| RVA+NoV | 5 | 2 |  |  |  |
| NoV+AdV | 1 | 2 |  |  |  |
| RVA+AdV+NoV | 1 |  |  |  |  |
| RVA+AdV+SaV |  | 1 |  |  |  |
| Total | 14 | 21 |  |  |  |
