# Supplementary material for: Viral Etiology of Acute Gastroenteritis Among Forcibly Displaced Myanmar Nationals and Adjacent Host Population in Bangladesh
Source: J Infect Dis. 2021 Sep 29;224(Suppl 7):S864–72. doi: 10.1093/infdis/jiab466 (PMC8687051; doi:10.1093/infdis/jiab466)
Supplement: jiab466_suppl_Supplementary_Table_S2 [file jiab466_suppl_supplementary_table_s2.docx]

| **Table S2: Prevalence of gastrointestinal bacterial pathogens among different age groups of FDMN and AHP** | | | | | | | | | | |
| --- | --- | --- | --- | --- | --- | --- | --- | --- | --- | --- |
|  |  |  |  |  |  |  |  |  |  |  |
|  | **FDMN-bacterial infections** | | | | | **AHP-bacterial infections** | | | | |
| **Age group** | **No. of samples** | **Prevalence (n%)** | **P values** | **OR** | **95% CI** | **No. of samples** | **Prevalence (n%)** | **P values** | **OR** | **95% CI** |
| **≤2 years** | 44 | 4 (9) | 0.11 | 0.331 | 0.099-1.099 | 62 | 7 (11.3) | <0.05 | 0.276 | 0.097-0.782 |
| **>2-5 years** | 5 | 0 | - | - | - | 2 | 0 | - | - | - |
| **>5-18 years** | 11 | 0 | - | - | - | 7 | 4 (57.1) | <0.05 | 6.933 | 1.406-34.19 |
| **>18 years** | 40 | 13 (32.5) | 0.001 | 6.741 | 2.008-22.63 | 29 | 8 (27.6) | 0.26 | 2.078 | 0.736-5.864 |
